# Report of a Case of Idiosyncracy to Quinine and Urea Hydrochloride

**Published:** 1917-12

**Authors:** Collier F. Martin

**Affiliations:** Philadelphia, Pa.


### REPORT OF A CASE OF IDIOSYNCRACY TO QUININE AND UREA HYDROCHLORIDE.

**By Collier F. Martin, M.D., Philadelphia, Pa.**

Martin reports a case of toxic symptoms appearing in a patient from an injunction of 3m. of a 10 per cent. solution of quinine and urea hydrochloride. The symptoms compalined ot by the patient were swelling of the hands and feet, and numbness of the extremities. For a few hours there was some difficulty in respiration, associated with a tendency to fainting and some nervous perturbation. Later there developed an urticarial rash, covering the entire body, associated with intense itching. The attack subsided in about two days, leaving the patient with no alarming symptoms. The patient has had two previous experiences, and certainly should have informed her physician of her susceptibility. The case is cited simply to note one of the complications which may occur when using this drug.

